# Hyperspectral Leaf Reflectance Detects Interactive Genetic and Environmental Effects on Tree Phenotypes, Enabling Large‐Scale Monitoring and Restoration Planning Under Climate Change

**DOI:** 10.1111/pce.15263

**Published:** 2024-11-04

**Authors:** Jaclyn P. M. Corbin, Rebecca J. Best, Iris J. Garthwaite, Hillary F. Cooper, Christopher E. Doughty, Catherine A. Gehring, Kevin R. Hultine, Gerard J. Allan, Thomas G. Whitham

**Affiliations:** ^1^ Department of Biological Sciences Northern Arizona University Flagstaff Arizona USA; ^2^ School of Earth and Sustainability Northern Arizona University Flagstaff Arizona USA; ^3^ Center for Adaptable Western Landscapes Northern Arizona University Flagstaff Arizona USA; ^4^ School of Informatics, Computing and Cyber Systems Northern Arizona University Flagstaff Arizona USA; ^5^ Department of Research, Conservation and Collections Desert Botanical Garden Phoenix Arizona USA

**Keywords:** common garden, gene by environment interaction, heritability, hyperspectral reflectance, leaf spectra, phenotypic plasticity, *Populus fremontii* (Fremont cottonwood), PRI

## Abstract

Plants respond to rapid environmental change in ways that depend on both their genetic identity and their phenotypic plasticity, impacting their survival as well as associated ecosystems. However, genetic and environmental effects on phenotype are difficult to quantify across large spatial scales and through time. Leaf hyperspectral reflectance offers a potentially robust approach to map these effects from local to landscape levels. Using a handheld field spectrometer, we analyzed leaf‐level hyperspectral reflectance of the foundation tree species *Populus fremontii* in wild populations and in three 6‐year‐old experimental common gardens spanning a steep climatic gradient. First, we show that genetic variation among populations and among clonal genotypes is detectable with leaf spectra, using both multivariate and univariate approaches. Spectra predicted population identity with 100% accuracy among trees in the wild, 87%–98% accuracy within a common garden, and 86% accuracy across different environments. Multiple spectral indices of plant health had significant heritability, with genotype accounting for 10%–23% of spectral variation within populations and 14%–48% of the variation across all populations. Second, we found gene by environment interactions leading to population‐specific shifts in the spectral phenotype across common garden environments. Spectral indices indicate that genetically divergent populations made unique adjustments to their chlorophyll and water content in response to the same environmental stresses, so that detecting genetic identity is critical to predicting tree response to change. Third, spectral indicators of greenness and photosynthetic efficiency decreased when populations were transferred to growing environments with higher mean annual maximum temperatures relative to home conditions. This result suggests altered physiological strategies further from the conditions to which plants are locally adapted. Transfers to cooler environments had fewer negative effects, demonstrating that plant spectra show directionality in plant performance adjustments. Thus, leaf reflectance data can detect both local adaptation and plastic shifts in plant physiology, informing strategic restoration and conservation decisions by enabling high resolution tracking of genetic and phenotypic changes in response to climate change.

## Summary statement

By growing the same locally adapted populations in reciprocal common gardens, we found that leaf reflectance can detect both genetic identity and phenotypic plasticity in Fremont cottonwood, an important riparian foundation species.

## 1 Introduction

Because ecosystem structure and function are often highly dependent on the traits of component plant species, understanding the effects of changing environmental conditions on plant phenotypes is critical as global climate change continues (Nicotra et al. 2010; Anderegg et al. 2019; Bonamour et al. 2019; Anderson and Song 2020; Brooker et al. 2022; Anderegg 2023). Plant phenotype is jointly determined by genetic and environmental factors, and it is important to understand which traits are sensitive to environmental change and which are relatively stable across different environments. Unfortunately, quantifying functional traits across multiple environments over large time periods is both time and resource intensive. In contrast to morphological, physiological, and chemical assays, leaf reflectance data can potentially provide non‐destructive and efficient screening of many plant traits at multiple genetic (Meireles et al. 2020; Stasinski et al. 2021) and geographic scales (Stein et al. 2014; Dungey et al. 2018; Moran et al. 2023). Hyperspectral leaf reflectance, which is the measurement of light energy reflected across a large number of individual wavelengths, can efficiently capture many separate dimensions of trait variation, and can be measured on the ground as a feature of individual leaves, or via drones, aircraft, or satellite as a feature of the tree canopy. Several studies have shown that leaf spectra may more accurately predict plant identity than models using molecular (Ballesta et al. 2022), physiological (Yan et al. 2021), or leaf economic and functional traits (Cavender‐Bares et al. 2016; Villa et al. 2021).

Leaf reflectance spectra can be useful in studies of trait differentiation because they are highly multivariate, with different wavelengths acting as proxies for specific chemical or physiological traits or as indicators of plant health. For example, spectra in the visible light region are predictive of concentrations of pigments such as chlorophyll, carotenoids, and xanthophylls (Knipling 1970). The visible region (Carter 1993) or the transition region between visible red to near infrared (NIR) can indicate plant stress (Curran, Dungan, and Gholz 1990; Peñuelas and Filella 1998). NIR wavelengths can predict anatomical and morphological traits such as the cellular structure of mesophyll (Woolley 1971) and carbohydrate content (Das et al. 2018; Ely et al. 2019). Shortwave infrared (SWIR) reflectance can predict the concentrations of chemicals such as tannins (Lehmann et al. 2015) and phenolic glycosides (Couture et al. 2016), as well as water content (Gates et al. 1965). These traits can indicate different physiological adaptations or acclimations to environmental conditions, and also have ecosystem‐wide impacts on processes such as nutrient cycling, drought resistance, plant growth, and source‐sink balance (Ely et al. 2019). Chemical traits, for example, can shape the composition of associated communities of insects and pathogens (Levin 1976; Richards et al. 2015; Cosmo et al. 2021; Fernandez‐Conradi et al. 2022).

Predicting future plant phenotypes requires us to quantify how plant traits depend on genetic identity versus responses to new environmental conditions (i.e., phenotypic plasticity). Plant genotypes also often differ in their environmental responses, producing gene by environmental interactions (Via and Lande 1985; Nicotra et al. 2010). Previous studies on the determinants of leaf reflectance spectra in forest trees have largely focused on quantifying the genetic component and have found differences in spectra at the genotype and population scales (Cavender‐Bares et al. 2016; Blonder et al. 2020, 2022; Stejskal et al. 2023). However, we know much less about the importance of these genetic effects relative to plastic responses to rapidly changing temperatures, which is a major constraint on our ability to predict future trait distributions under climate change. Here, we address this gap by evaluating genetic and environmental influences on leaf reflectance across a steep climatic gradient in *Populus fremontii* S. Watson (Fremont cottonwood), a foundation tree species of riparian ecosystems with multiple ecotypes (Bothwell et al. 2023) across the Southwest region of the United States.

Fremont cottonwood ecotypes occupy diverse climate niches, ranging from hot sea‐level habitats in southern Arizona to cooler high‐elevation areas on the Colorado Plateau, and show substantial intraspecific genetic divergence among populations of each ecotype (Grady et al. 2011; Cushman et al. 2014; Ikeda et al. 2017; Cooper et al. 2019). The populations studied here have evolved locally adapted traits consistent with climate‐driven selection (Hultine et al. 2020; Blasini et al. 2021; Cooper et al. 2022; Moran et al. 2023), but many of these traits also show substantial plasticity. For example, interactions between genetic identity and growing environment can shape leaf phenology (Cooper et al. 2019), leaf morphology and decomposition (Jeplawy et al. 2021), and phytochemistry (Eisenring et al. 2022). Since many of these traits are often highly predictable from leaf reflectance spectra (Grzybowski et al. 2021), spectra should provide a more efficient way to quantify gene by environmental interactions in these traits over larger areas and better temporal resolution than direct trait assessments, which can be time consuming and costly. If spectra can reliably detect genotype and population level differences regardless of growing environment, they could also be used to screen trees for specific advantageous trait values or specific types of plastic responses. Furthermore, as foundation species (Ellison et al. 2005) cottonwoods largely define many riparian ecosystems that are increasingly threatened by extreme temperatures and drought (Moran et al. 2023) and projected to suffer major declines in their geographic distribution (Bothwell et al. 2021). Cottonwoods support diverse communities that differ among individual tree genotypes, populations, and ecotypes, due to their multivariate trait phenotypes (Holeski et al. 2012; Lamit et al. 2015; Whitham et al. 2020; Bothwell et al. 2023). As such, novel methods for predicting future phenotypes could also help predict impacts on dependent communities and ecosystem processes in this and other forest ecosystems.

**Figure 1:**
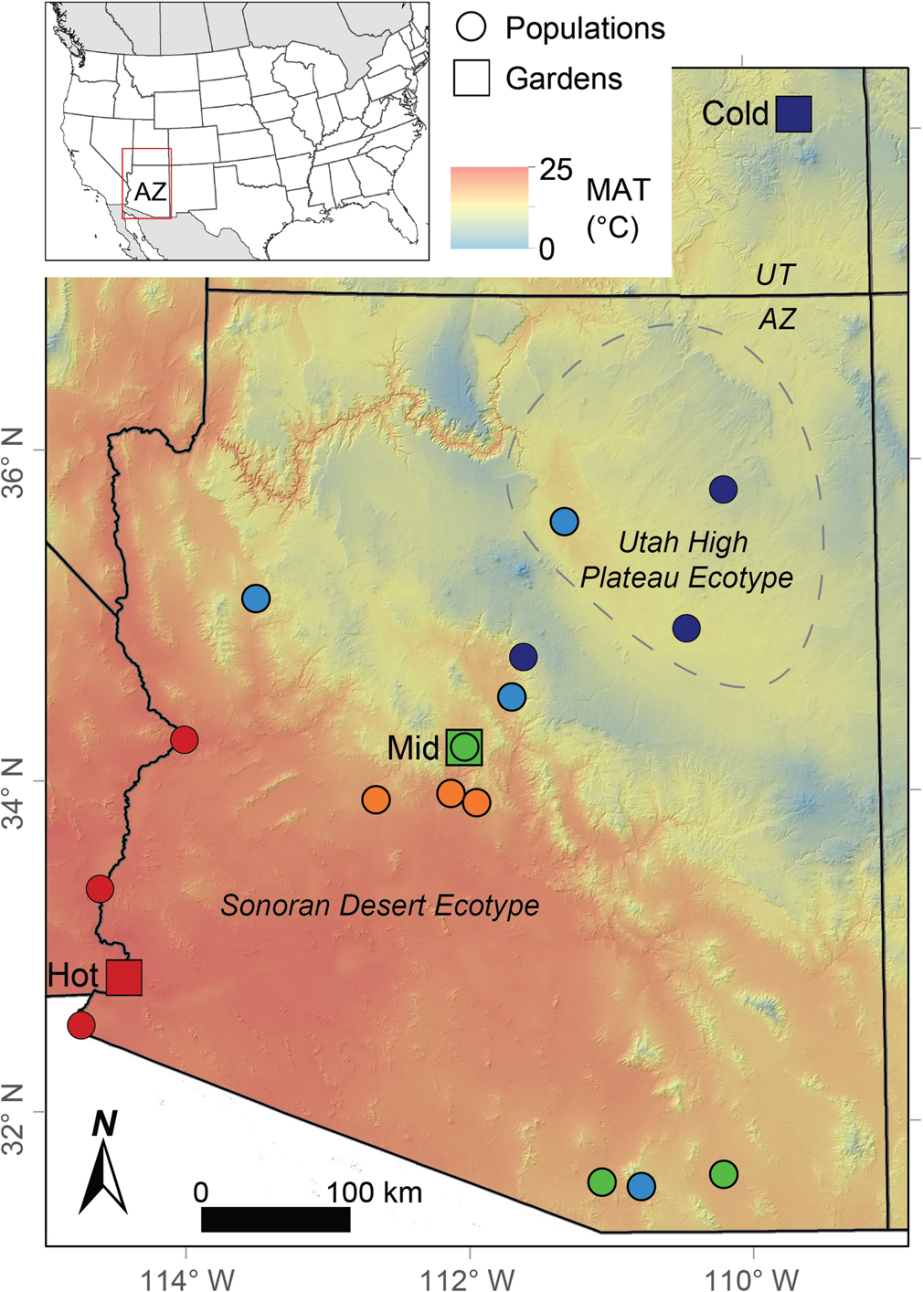
Locations of population home sites and the three common gardens (Cold, Mid and Hot) along a gradient in mean annual temperature set by increasing elevation from southwest to northeast. The ‘Mid’ common garden is at intermediate elevation and temperature representing the centre of the species' climate range in Arizona. As indicated by the dashed line, the three northeastern populations fall within the Utah High Plateau ecotype, whereas the remaining populations are part of the Sonoran Desert ecotype (Bothwell et al. 2023). Climate data from WorldClim 2 (Fick and Hijmans 2017).

**Figure 2:**
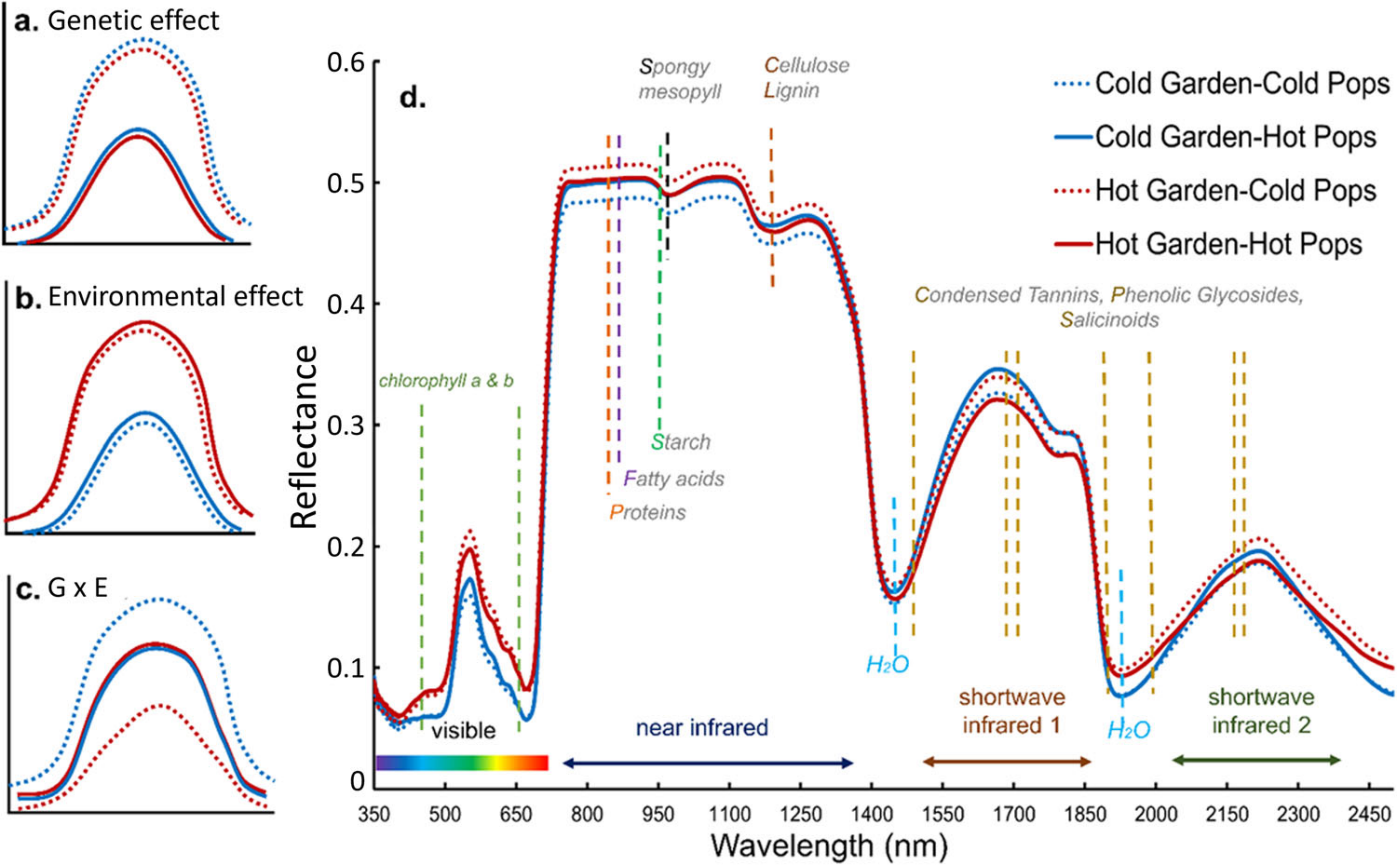
Spectral reflectance regions and hypothetical genetic and environmental effects. We present hypothetical reflectance scenarios which show: (a) a genetic effect in which spectral amplitudes differ across source populations, (b) an environmental effect in which spectral amplitudes differ between common garden climates and (c) an interactive (g x e) effect where the degree of genetic differentiation depends on the growing environment. We also show (d) actual averaged leaf reflectance measurements from the three populations from the coldest source sites and the three populations from the hottest source sites, growing in the Cold and Hot common gardens. Reflectance units are a ratio from 0 to 1, where 1 is 100% reflectance. Vertical dashed lines indicate wavelengths associated with several important biochemical and morphological traits as documented in the following references: chlorophyll (Knipling 1970), mesophyll (Woolley 1971), starches (Das et al. 2018), sugars and proteins (Ely et al. 2019), tannins (Lehmann et al. 2015), phenolic compounds (Couture et al. 2016) and water content (Gates et al. 1965).

To test whether leaf reflectance can detect both genetic and plastic differences in this species, we sampled leaf reflectance spectra from Fremont cottonwood clones collected from 12 populations and reciprocally transplanted to three common gardens in cold, moderate (henceforth ‘mid’, intermediate in elevation and temperature), and hot locations (Figure 1). Replicated common garden experimental designs are particularly powerful for parsing whether trait variation is due to underlying genetics, environmental factors, or gene by environment interactions. Using wavelengths from 500 to 2300 nm, which includes spectral regions known to correlate with morphological and chemical leaf traits (Figure 2a), we compared trees within and across gardens. For each hypothesis, we combined approaches using the full spectra and approaches using specific wavelengths to calculate commonly used indices of plant physiology and photosynthetic efficiency (Gamon, Serrano, and Surfus 1997; Eitel et al. 2006; Letts et al. 2008).

First, we hypothesized **(H1)** that leaf spectra would show heritable genetic effects across populations and genotypes (Figure 2a). We investigated this by (a) testing whether multivariate leaf spectra could accurately identify populations and genotypes growing within any common environment, and (b) quantifying the heritability of common spectral indices. Second, we hypothesized **(H2)** that growing environment would shift leaf reflectance via phenotypic plasticity (Figure 2b), and that the extent of plasticity would vary across populations, producing gene by environment (g x e) interactions (Figure 2c). We investigated this by (a) testing for genetic, environmental, and g x e effects on multivariate spectra, (b) comparing the degree of environmental effects among spectral regions, which are associated with different traits, and (c), by testing for g x e effects on spectral indices. Finally, we predicted **(H3)** that indicators of altered plant physiology or stress (such as reduced photosynthetic efficiency) should increase with the magnitude of environmental change between their transferred garden location and the local conditions to which populations are adapted. We investigated this by quantifying the difference in mean annual maximum temperature between home sites and common gardens, and testing whether this transfer distance could predict variation in spectral indices of plant physiology. Collectively, tests of these hypotheses will determine how leaf reflectance spectra indicate ways that plant physiology and genetic diversity within and among populations respond to ongoing and projected future climate changes. If leaf spectra can accurately indicate plant genotype and phenotypes across spatial and temporal scales, this could significantly improve restoration planning and ecosystem monitoring in a region simultaneously experiencing ongoing megadrought (Williams, Cook, and Smerdon 2022) and extreme temperatures (McKinnon, Poppick, and Simpson 2021). If found to be broadly applicable, this approach could assist worldwide efforts to mitigate global change impacts on vegetation (IPCC 2022).

## 2 Materials and Methods

### 2.1 Study Sites

In this study we used three Fremont cottonwood common gardens established in 2014 in cold, hot and moderate locations along a steep elevation gradient in Arizona and Utah, USA (Figure 1). Cuttings from multiple genotypes within each of 16 wild populations representing two ecotypes, the Sonoran Desert ecotype, and the Utah High Plateau ecotype (Bothwell et al. 2023) were harvested and reared at the Northern Arizona University research greenhouse for 9 months, then planted in the common gardens in a randomized block design with 2 m spacing. Supplemental water was provided via drip irrigation to each individual tree at the Cold and Mid gardens, and via flood irrigation at the Hot garden during the growing season. More information on garden design and establishment is available in Cooper et al. (2019). For this study, we included 12 populations spanning the temperature and elevational ranges of Fremont cottonwood in Arizona (see Supporting Information S1: Table S1 for GPS coordinates). In each garden we sampled 3–4 replicate trees for each of the same 4–12 genotypes per population, for a total of 74 genotypes. Spectral measurements were also collected at 10 of the original wild populations (henceforth called ‘home sites’) where the common garden cuttings were harvested in 2014 (Figure 1).

### 2.2 Spectral Data

In summer of 2020, we collected leaf spectral data in the three experimental common gardens. We sampled in the Hot garden 9–15, the Mid garden May 25–29, and the Cold garden June 22–25. All days were clear and temperatures were similar throughout each sampling week. The temporal separation between gardens was designed to match the differences in leaf phenology between these gardens (Cooper et al. 2019). Three to five fresh leaves were collected per tree and immediately placed in a cooler. Readings were taken within 2 h of harvest. To standardize for variation within the canopy, leaves were collected on either the west or east side of the tree at breast height (~1.5 m). We standardized leaf age by only sampling mature, fully expanded ‘early’ leaves formed in the buds set the previous fall (Critchfield 1960; Neuwirthová et al. 2021). Spectral measurements were taken with a handheld spectrometer (ASD Fieldspec 3, Malvern Panalytical) and ASD leaf clip attachment fitted with an optical black and white standard. The spectrometer is equipped with three internal sensors which measure reflectance values from 350 to 2500 nm with resolutions of 3 nm at 700 nm and 10 nm at 1400 and 2100 nm (Figure 2). Care was taken to measure one bilateral half of the leaf tissue on the adaxial surface avoiding the midrib. After allowing the ASD to warm up for 30 min, we measured reflectance using the optical black standard. We obtained the spectral data for each leaf by averaging three measurements at different locations on the leaf, with each measurement itself the average of five internal readings. We recalibrated the spectrometer with the optical white standard approximately every 10 leaves. Before averaging to the tree level, anomalous samples with obviously spurious reflectance values (e.g., due to misalignment of the leaf clip) were removed (Burnett et al. 2021). A splice correction was applied to each reading to account for regions of sensor overlap (1000 and 1830 nm) using the *Prospectr* package (Stevens and Ramirez‐Lopez 2022) in R (R Core Team 2022). Following the recommendations presented in Burnett et al. (2021), we removed the potentially noisy data below 500 nm and above 2300 nm, but did not use data pre‐processing or transformation techniques aside from splice correction and removal of obviously erroneous reflectance readings before statistical analysis.

Spectral data were collected at the home sites in June 2020. At each site, we revisited the original mother trees that supplied the cuttings for the gardens, collected ten fresh leaves per tree, and analyzed the spectra using the same protocol implemented for common garden trees. Two populations (Sonoita Creek and Keams Canyon) were inaccessible due to travel and U.S. border restrictions. The wild site data serve as a baseline to compare spectral changes from each genotype in its home environment to its clonal offspring transplanted to a common garden.

### 2.3 Statistical Analyses

#### 2.3.1 H1 ‐ Genetic Effects and Heritability of Leaf Reflectance Spectra

We tested for genetic effects on multivariate spectra within a growing environment using partial least squares discriminant analysis (PLS‐DA). Categories included genotype, population, and environment (common garden). PLS‐DA is a supervised method routinely used to assess large multivariate datasets such as leaf reflectance. It functions by classifying samples into groups based on the latent structure of predictor and response variables. PLS‐DA was implemented in R using the *caret* (Kuhn 2008) and *vegan* (Oksanen et al. 2022) packages as well as the protocol outlined by Burnett et al. (2021). To determine whether to use all leaves for each tree or an average of leaves we compared preliminary PLS‐DA models for each. We found that using all leaves provided slightly higher predictive accuracy and included leaf‐level data for PLS‐DA modelling. We did not correct for time of day as previous studies have shown that reducing variation in wavelengths to control for time reduced the accuracy of predictive models (Barnes et al. 2017; Ely et al. 2019).

Data points were subset according to garden, population, or genotype to ensure even contribution from each category, then randomly assigned to calibration and validation datasets composed of 80% and 20% of the data, respectively. Model training was performed using 10‐fold cross validation repeated three times. This means splitting the calibration data set into 10 random groups and using each group as the test set to be predicted with the model. The optimal number of components to include in the model was identified by comparing models with increasing components over 100 iterations and was based on the highest kappa value. In classification models, kappa shows how well a classifier performs compared to a random assignment. Values less than or equal to zero indicate a poor classifier, while positive values indicate agreement. Model performance was estimated based on the kappa and accuracy values of the validation (test) set. Influential predictor variables for each model were identified using the loading values to ascertain variables of importance (VIPs) for the predictive models. Finally, we constructed confusion matrices to show the percent of well classified observations, that is, the ratio of correctly assigned classifications over the total classifications. This measure is used to assess the performance of the PLS‐DA model by comparing training and validation similarity. We assessed classification accuracy within common garden environments and across all home sites.

In addition to testing for genotype and population effects on the full leaf reflectance spectra, we also calculated several spectral indices often inferred to indicate physiological strategies and/or some degree of stress either through chlorosis, low water content, or degradation of tissues. Formulas and references for each index are in Supporting Information S1: Table S2. These include indicators of photosynthetic pigment content, the chlorophyll index (CI; Gitelson and Merzlyak 1994) and carotenoid reflectance index (CRI; Gitelson, Keydan, and Merzlyak 2006), as well as an indicator of photosynthetic efficiency, the scaled photosynthetic reflectance index (sPRI; Gamon, Serrano, and Surfus 1997; Letts et al. 2008). We also calculated an indicator of vegetation greenness, the normalized difference vegetation index (NDVI; Tucker 1979), and two indicators of water content, the normalized difference water index (NDWI; Gao 1996), and maximum difference water index (MDWI; Eitel et al. 2006). To examine the strength of genetic effects on these indices we quantified the heritability of each index within each environment. In a common environment, broad sense heritability can be quantified as the proportion of total phenotypic variance (*V_P_*) that is due to genotypic variance (*V_G_*). We calculated *V_G_*/*V_P_* using a mixed model implemented in *lme4* (Bates et al. 2015) for each common garden. We extracted the variance components for *V_G_* = the random effect of genotype and *V_P_* = *V_G_* + V_resid_. Using 1000 iterations of each model, we calculated both means and 95% confidence intervals for heritability estimates across all genotypes (collection‐wide) and across genotypes nested within populations (hierarchical) as in Evans et al. (2016).

#### 2.3.2 H2—Environmental Modification of Leaf Reflectance Across Wavelengths and Indices

Moving from single‐garden to multi‐garden comparisons, we tested the influence of genotype, population, garden, and their interactions on leaf spectra by conducting a three‐way permutational analysis of variance (PERMANOVA). The PERMANOVA test was completed using the ‘adonis2’ function from the *vegan* package (Oksanen et al. 2022) with the input being a distance matrix using Bray‐Curtis dissimilarity in the ‘vegdist’ function. We assessed term significance by margin and repeated the model for 999 iterations. Following PERMANOVA, we assessed the homogeneity of group dispersions using the ‘permdist’ function.

To examine how different regions of the reflectance spectra are influenced by genetic versus environmental factors, we plotted the difference in reflectance for each population between their home site and each common garden environment. Finally, we again used our set of spectral indices to examine phenotypic responses across growing environments (i.e., reaction norms of spectral traits). We used linear mixed models to fit fixed effects of common garden environment and random effects of population and genotype, and then plotted population x environment means to examine environmental effects on each index. We also compared these to values of the indices for the populations at their home sites.

#### 2.3.3 H3—Transfer Distances From Home Climates

Given that these populations show evidence of local adaptation, with maximum survival and growth in their home climates (Cooper et al. 2019, 2022), we tested whether genotypes transferred a greater temperature difference from home showed spectral signals of greater physiological adjustment or higher stress (e.g., reduced photosynthetic efficiency). To do this we fit linear regressions of each of the spectral indices as a function of their transfer distance. We calculated the difference in the mean annual maximum temperature (MAMT C°) between each population's home site and each common garden, where positive values of transfer distance indicate moving a population to a hotter temperature than its home climate and negative values indicate moving a population to a colder temperature than its home climate. MAMT for each home and common garden site was obtained from WorldClim (Fick and Hijmans 2017).

## 3 Results

### 3.1 H1—Leaf Reflectance Spectra Differ Among Genotypes and Populations

**Figure 3:**
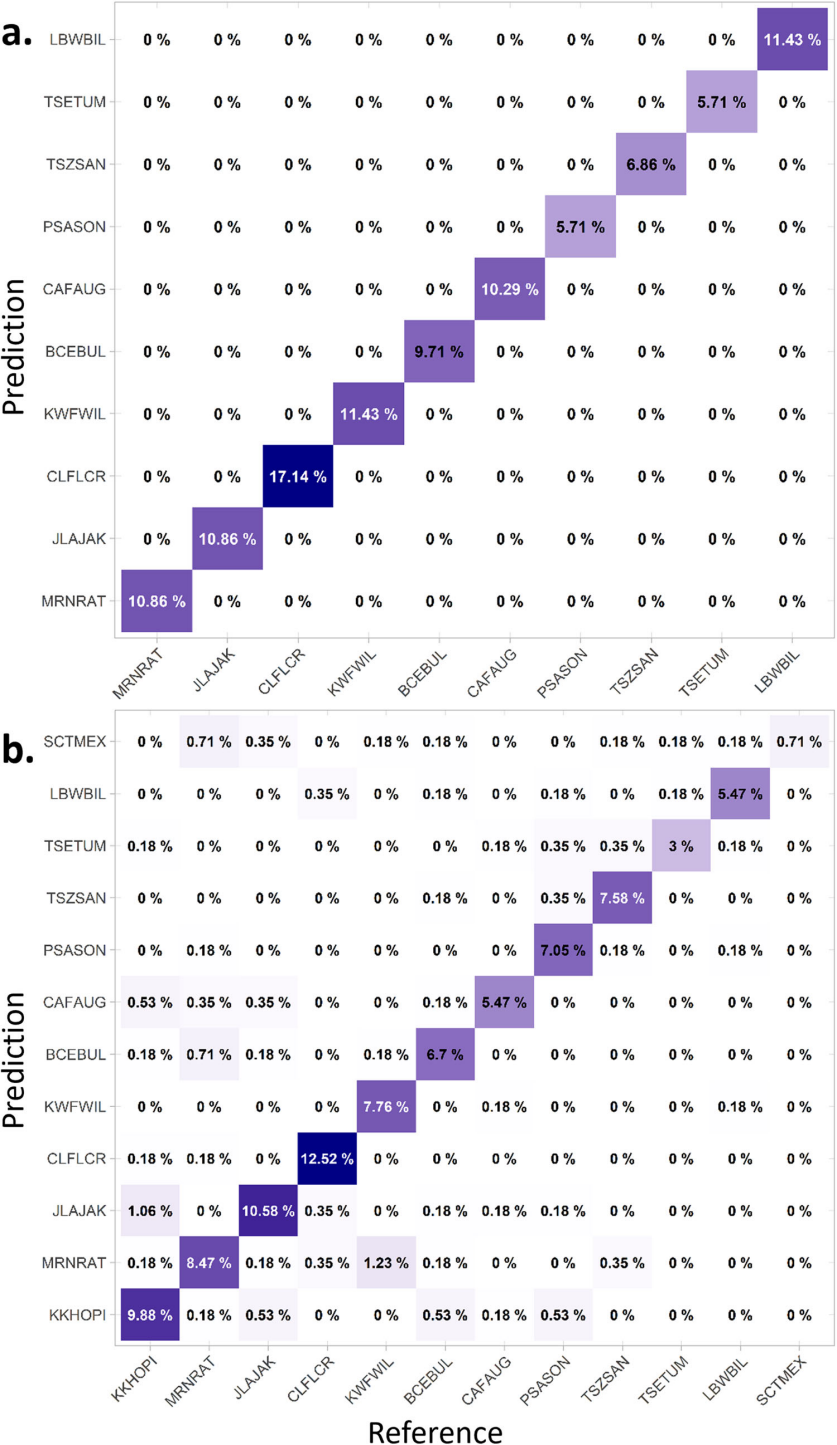
Confusion matrices of partial least squares discriminant analyses (PLS‐DA) validation (test) results between genotypes, populations, and environments. Matrices show the percent of correct classification between the reference (training) data and the prediction (test) data. The diagonal line indicates correct classification for the category while areas adjacent to the diagonal indicate a false classification by the model. Populations are ordered by mean annual temperature at the home site. We show (a) predictions of population identity for populations at source home sites (100% accuracy) and (b) predictions of population identity across all common gardens pooled together (86% accuracy, sum of the diagonal values). Note that two populations (KKHOPI, SCTMEX) could not be resampled at home due to access restrictions. [Color figure can be viewed at wileyonlinelibrary.com]

Within a common garden, we found that populations were clearly predictable (87%–98% accuracy) and genotypes less so (30%–43% accuracy for the 74 genotypes) using PLS‐DA (Table 1). In comparison, population was predictable with 100% accuracy among the home sites (Figure 3a), whereas pooling the data from all gardens had the lowest predictive accuracy (Figure 3b). This illustrates that comparing spectra among wild populations, where both genetic identity and local environmental conditions may differ, leads to maximal differentiation in leaf reflectance. Conversely, pooling data from multiple common gardens adds environmental noise to each genotype due to plastic effects on spectra (see Supporting Information S1: Figure S1 for plots of the full spectra in each environment). However, even across the environmental variation of the three common gardens pooled together we could still identify populations with 86% accuracy, which was similar to within the Mid common garden.

**Table 1:** PLS‐DA results for predicting genotype, population or environment identity using leaf hyperspectral data.

Classification Unit	Sites	Accuracy (sd) [95% CI]	Kappa (sd)	Components
Genotype	All gardens	0.24 (0.04) [0.21, 0.28]	0.23 (0.05)	10
	Cold	0.43 (0.09) [0.34, 0.51]	0.40 (0.07)	10
	Mid	0.30 (0.06) [0.26, 0.37]	0.30 (0.05)	10
	Hot	0.44 (0.08) [0.36, 0.53]	0.43 (0.08)	10
Population	All gardens	0.86 (0.04) [0.82, 0.88]	0.84 (0.04)	77
	Cold	0.98 (0.01) [0.97, 0.98]	0.99 (0.02)	60
	Mid	0.87 (0.01) [0.82, 0.91]	0.86 (0.01)	67
	Hot	0.94 (0.01) [0.89, 0.97]	0.93 (0.02)	61
	Home	1 (0.01) [0.96, 1.00]	0.97 (0.02)	36
Garden	All gardens	1 (0.02) [0.95, 1.00]	0.98 (0.02)	10

*Note:* Separate PLS‐DA tests were used to predict genotype and population identity within each common garden as well as among all gardens. ‘Site’ indicates in which environment the model is predicting a classification unit, for example, predicting genotype identity in the Cold garden. Only genotypes present in all three common gardens with an *n* ≥ 3 were included for the ‘All gardens’ analysis. We report the validation (test) results as: ‘Accuracy’ with standard deviation with a 95% confidence interval, ‘Kappa’ with standard deviation, and the number of components used for each PLS‐DA. Accuracy indicates the percent of correct classifications where 1.0 indicates 100% correct predictions based on spectra and 0 indicates all incorrect predictions. ‘Kappa’ is a chance‐corrected metric which shows both the accuracy and reliability of classifications. Population identity was predicted within each common garden, among all common gardens and among home sites. To predict populations from all common gardens, only populations present in every garden were used. Lastly, we used PLS‐DA to predict environment identity, that is, which common garden a leaf came from.

Just as prediction accuracies were higher for the 12 populations than the 74 genotypes (Table 1), heritability estimates were higher when considering genetic variation among all genotypes in each garden rather than only within populations (Table 2). These collection‐wide heritability values were significant for all indices in the Mid common garden and three to four of the six indices in the other gardens. However, half of the indices (CI, CRI and NDVI) also had significant within‐population heritability in at least one of the common gardens, and all three were significant in the Mid garden (Table 2). Of those spectral heritability estimates that were significant, genotypic variation accounted for 10%–23% of the variation within populations and 14%–48% of the variation among all genotypes regardless of population.

**Table 2:** Broad‐sense heritability estimates for each spectral index for each common garden, with 95% confidence intervals from 1000 bootstraps in parentheses.

	*H* ^2^ Hierarchical (within populations)			*H* ^2^ Collection‐wide (across all genotypes)		
Index	Cold	Mid	Hot	Cold	Mid	Hot
CI	0.11 (0, 0.24)	**0.18 (0.08, 0.29)**	**0.23 (0.01, 0.44)**	**0.28 (0.10, 0.39)**	**0.32 (0.19, 0.43)**	**0.45 (0.14, 0.64)**
CRI	**0.12 (0.07, 0.22)**	**0.13 (0.04, 0.24)**	0.00 (0, 0.06)	**0.48 (0.28, 0.61)**	**0.42 (0.26, 0.55)**	0.11 (0, 0.24)
sPRI	0.00 (0, 0)	0.00 (0, 0.06)	0.09 (0, 0.22)	0.03 (0, 0.22)	**0.14 (0.02, 0.23)**	**0.38 (0.12, 0.57)**
NDVI	0.00 (0, 0.34)	**0.10 (0.02, 0.21)**	0.00 (0, 0.03)	**0.34 (0.06, 0.62)**	**0.38 (0.2, 0.49)**	0.17 (0, 0.33)
NDWI	0.11 (0, 0.33)	0.00 (0, 0.11)	0.12 (0, 0.27)	0.13 (0, 0.33)	**0.31 (0.1, 0.45)**	**0.34 (0.19, 0.42)**
MDWI	0.13 (0, 0.29)	0.04 (0, 0.2)	0.02 (0, 0.25)	0.15 (0, 0.3)	**0.30 (0.14, 0.4)**	**0.26 (0.04, 0.47)**

*Note:* Cases where the confidence interval does not include 0 are bolded to show significant heritability. As described in Evans et al. (2016), *H* ^2^ hierarchical accounts for and removes population variance from the heritability equation (i.e., genetic variation within populations), whereas *H* ^2^ collection‐wide calculates heritability across all genotypes regardless of population (i.e., all genetic variation within and among populations). Abbreviations are: chlorophyll index (CI), carotenoid reflectance index (CRI), scaled photochemical reflectance index (sPRI), normalized difference vegetation index (NDVI), normalized difference water index (NDWI), and maximum difference water index (MDWI).

### 3.2 H2—Environmental and Gene‐by‐Environment Effects Modify Leaf Reflectance Spectra

In agreement with our hypothesis that leaf spectra are jointly determined by genetic and environmental effects, PERMANOVA tests using full spectra (500–2400 nm) showed significant main and interactive effects (Table 3). Across all wavelengths on average, environment affected reflectance in ways that were population‐specific. This included both across common gardens increasing in temperature (Table 3a, note the exclusion of the Cold common garden because not all of the hot populations had survived there) and between home sites and the Mid‐elevation garden at the centre of the climate gradient (Table 3b).

**Table 3:** PERMANOVA results showing main effects of population and environment and their interactions.

Model	Source	Df	Mean Sq	*F*	*R* ^2^	*p*
a.	Environment (Hot vs. Mid garden)	1	0.1102	62.2	0.030	< 0.001
	Population	11	0.1941	10.0	0.052	< 0.001
	Environment x population	11	0.5101	30.6	0.137	< 0.001
b.	Environment (Home vs. Mid garden)	1	0.1174	203.5	0.296	< 0.001
	Population	9	0.0068	11.7	0.153	< 0.001
	Environment x population	9	0.0033	5.7	0.075	< 0.001

*Note:* We tested for (a) spectral effects of growing environment between the Mid and Hot common gardens, simulating a warming effect between two controlled environments, and (b) effects of moving trees from their environmentally different home sites to the common growing conditions of the Mid garden.

**Figure 4:**
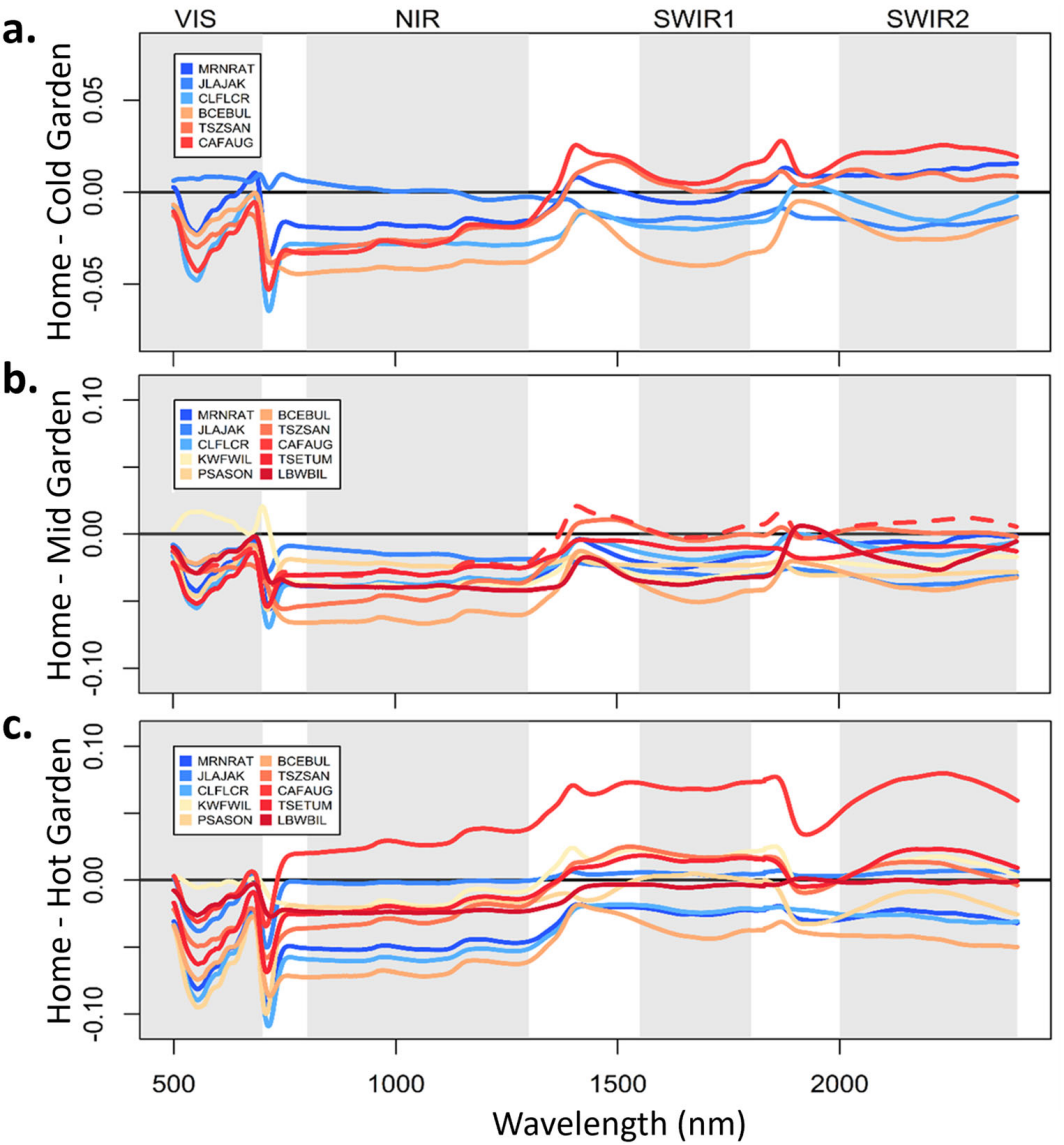
Differences on average leaf reflectance values between each of the (a) Cold, (b) Mid and (c) Hot gardens and home sites. Reflectance units are a ratio from 0 to 1, where 1 is 100% reflectance. Values of 0 indicate no change in reflectance between the garden and home site. Positive values indicate a decrease in reflectance in the common garden and negative values show an increase in reflectance in the common garden. The dashed line in panel b represents the wild population growing adjacent to this garden (CAFAUG). Populations are coloured by home site MAMT (Mean Annual Maximum Temperature) with cool colours indicating colder MAMT and warm colours indicating hotter MAMT.

Not only did environmental effects differ among populations, but they also varied across wavelengths. We found that moving trees from their home sites to common garden conditions generally increased their reflectance in the visible spectra (500–700 nm), leading to negative difference values in Figure 4. Because higher reflectance at these wavelengths has often been associated with reduced plant health, this may indicate stress responses to transplanting. This interpretation is validated by three cases where visible reflectance instead decreased slightly in the common garden or stayed the same (populations JLAJAK in the Cold garden and KWFWIL and CAFAUG in the Mid garden, Figure 4a,b). These are the common gardens closest to the home conditions for these populations, with CAFAUG originating from immediately outside the Mid garden. In contrast to the visible wavelengths, the decreased reflectance in the NIR and SWIR regions for CAFAUG transplanted to the Hot garden (Figure 4c) could indicate shifting values of leaf morphological and chemical traits.

**Figure 5:**
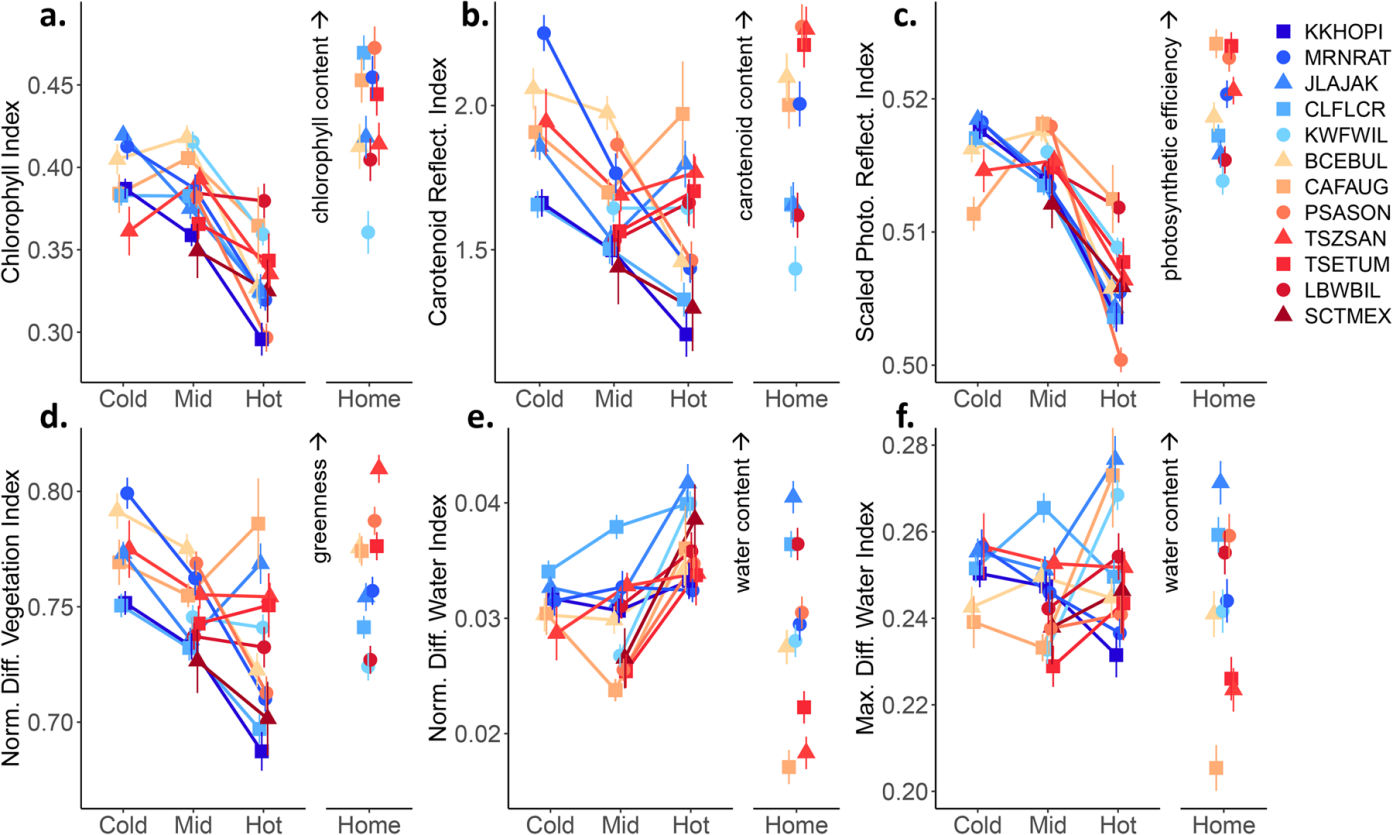
Reaction norms of common spectral indices showing the average index value for populations in each common garden environment (Cold, Mid, Hot). For all indices, the g x e interaction between population and garden environment was significant at *p* ≤ 0.001 (see Supporting Information S1: Table S3). We also show the spectral indices from each population at their home site as a baseline comparison (right column in each panel). Populations are coloured by home site MAMT (Mean Annual Maximum Temperature) with cool colours indicating colder MAMT and warm colours indicating hotter MAMT. Indices are: (a) chlorophyll index (CI), (b) carotenoid reflectance index (CRI), (c) scaled photochemical reflectance index (sPRI), (d) normalized difference vegetation index (NDVI), (e) normalized difference water index (NDWI) and (f) maximum difference water index (MDWI). Arrows indicate direction of increase in interpreted physical traits. Optimal values of most indices should be environmentally dependent except for sPRI, which is an indicator of photosynthetic efficiency. For sPRI, lower values suggest higher tree stress.

Focusing on spectral indices, across populations we also found significant environmental and genetic x environmental effects in their reaction norms (Figure 5). However, for the indices related to photosynthetic pigments and photosynthetic efficiency, there was a general decreasing trend for most populations with increasing garden temperature, either from the Cold garden to the Mid and Hot gardens, or from the Mid garden to the Hot garden. For the proxies of chlorophyll content (CI) and photosynthetic efficiency (sPRI), populations were relatively consistent in their shifts from Mid to Hot, but varied in their response to the Cold garden (Figure 5a,c). For carotenoid content (CRI) and greenness (NDVI), populations responded similarly to a temperature increase from the Cold to Mid gardens, but had very divergent responses to the Hot garden (Figure 5b,d). For the water content indices NDWI and MDWI, populations varied in their responses across all gardens (Figure 5e,f). Finally, most of the indices showed a similar amount of variation among home sites as they did among gardens (Figure 5), but photosynthetic efficiency tended to be the highest at home sites and lowest in the Hot garden (Figure 5c), likely indicating some reduced functioning at high temperatures.

### 3.3 H3—Spectral Indicators of Altered Plant Physiology or Stress Should Be Highest in Trees That Have Been Transferred the Greatest Climate Difference From Their Home Site

**Figure 6:**
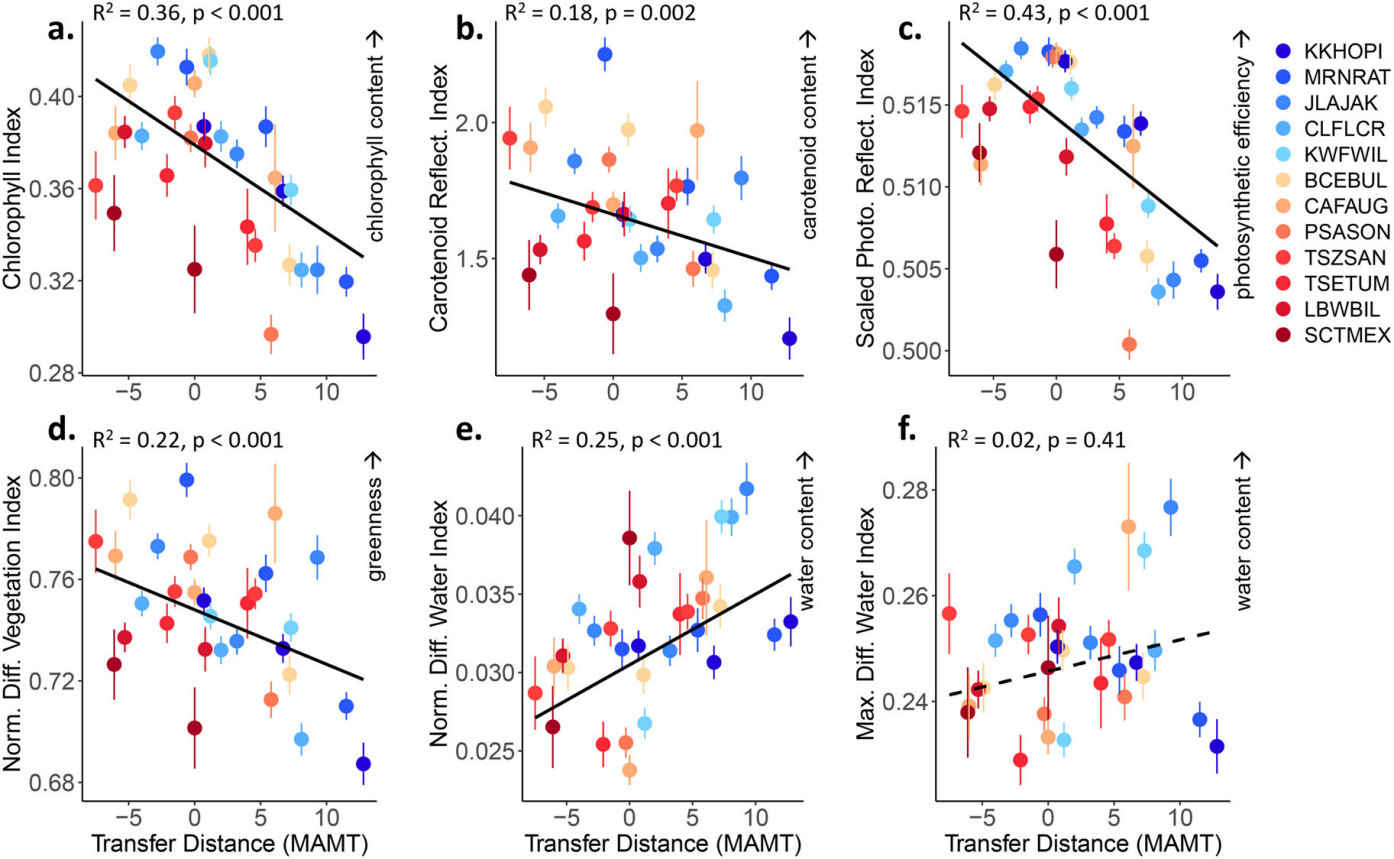
Linear regressions show population mean index values in each common garden (three points per population) as a function of transfer distance in Mean Annual Maximum Temperature (MAMT in ^**°**^C). The *x*‐axis is the difference of MAMT between home sites and common garden. Positive values of MAMT indicate transfer to a hotter temperature than a population's home climate and negative values indicate transfer to a colder environment. All regressions are significant at *p* ≤ 0.002 except for (f) MDWI, whether analyzed as a simple linear regression or as a mixed model regression with Population as a random factor. Populations are coloured by home site MAMT with cool colours indicating colder MAMT and warm colours indicating hotter MAMT. Full names of spectral indices are as in Figure 5. Arrows indicate direction of increase in interpreted physical traits. Optimal values of most indices should be environmentally dependent except for Scaled Photosynthetic Reflectance Index (sPRI), which is an indicator of photosynthetic efficiency. For sPRI, lower values suggest higher tree stress.

When analyzing spectral indices relative to a tree's change from home conditions, all indices were significantly related to MAMT transfer distance except for maximum difference water index (MDWI, Figure 6). Transfer to environments 5**°**C–10**°**C hotter than home conditions resulted in indications of decreased photosynthetic pigment content and greenness (Figure 6a,b,d). Transfer to hotter conditions also led to decreased sPRI, indicating decreased photosynthetic light use efficiency and CO_2_ uptake (Figure 6c). In contrast, transfer to colder environments than home decreased NDWI (Figure 6e), indicating more water stress in some hot populations moved to slightly colder locations than in cold populations moved to hot locations. Overall, transfer to hotter conditions appeared to cause shifts in physiological strategies that decreased photosynthetic efficiency more than water content. Transfer to colder conditions did not lead to any indicators of decreased chlorophyll or photosynthesis, but did appear to decrease water content. Thus, spectral indices show strong dependence of tree stress on the direction of temperature transfer, and different indices show distinct types of physiological adjustments or stress within trees moved in the same direction.

## 4 Discussion

### 4.1 Leaf Reflectance Is Jointly Shaped by Genetic and Environmental Factors

We present the first evidence that leaf reflectance can be used to detect both genetic and environmental effects for Fremont cottonwood (*Populus fremontii*) over its distribution along a steep climate gradient. Using a controlled comparison of populations adapted to divergent climatic regimes (Cooper et al. 2022) across multiple common gardens and home sites, we found that hyperspectral leaf reflectance data could identify wild populations with 100% accuracy. When we removed environmental variation using common gardens, we could still identify the genetic effects of population with 87%–98% accuracy and individual genotypes with 30%–43% accuracy (Table 1). Adding maximal environmental noise by pooling all common gardens still resulted in 86% accuracy for population identification. Although previous studies have shown that spectra can be used to identify different *Populus* species in a common garden (Deacon et al. 2017; Dmitriev et al. 2022), and can detect genetic variation within a *Populus* species in the wild (Blonder et al. 2020), these studies have not attempted to disentangle the relative influence of genetic and environmental effects on intraspecific trait variation. Here, we decompose the factors driving leaf reflectance in the wild, which is necessary to correctly interpret hyperspectral data to monitor and assess tree health, acclimation, and population genetic structure as climate change shifts environmental conditions across the landscape. We show that hyperspectral leaf reflectance can indeed be a valuable tool for helping to understand the large‐scale responses of foundation species and their associated ecosystems to rapid climate change. This includes identifying and tracking genetic composition and monitoring tree stress under changing conditions. With careful attention to the possible contributions of genetic and environmental components, including how far trees are from the historical climate conditions under which they evolved, our findings show that ecologists can leverage hyperspectral approaches to improve the conservation and restoration of wild species.

### 4.2 The Scale of Genetic Differentiation in Leaf Reflectance

So far, most research on spectral detection of taxonomic categories has either focused on detecting variation among or within species in the wild, where both genetics and environment may contribute (Madritch et al. 2014; Blonder et al. 2022; Czyż et al. 2023; Seeley et al. 2023a; D'Odorico et al. 2023), or detecting genotypes in highly controlled agricultural environments (Yao et al. 2004; Wang et al. 2010; Galán et al. 2020). In the wild, leaf spectra have been used to successfully identify evolutionary relationships among species (Meireles et al. 2020), with predictive accuracy increasing as phylogenetic scale increased from population to species to clade (Cavender‐Bares et al. 2016). Extending that finding to intraspecific variation while controlling for growing environment, we found that the accuracy of classification using PLS‐DA was higher at the population level than the genotype level (Table 1). Spectral indices were also more strongly differentiated across all populations than within populations (Table 2), but heritability estimates for some spectral indices were significant even within populations, explaining 10%–23% of the phenotypic variation (Table 2). These values fall within the range of heritability estimates for morphological and phenological traits in this species (Cooper et al. 2022) and support our first hypothesis that spectra can detect genetic differences at both levels. This finding is not surprising given that spectroscopy can predict a wide range of plant traits in *Populus* species that are often genetically differentiated, including phytochemistry (Rubert‐Nason et al. 2013; Couture et al. 2016; Kyaw et al. 2022), photosynthetic capacity (Kyaw et al. 2022), chlorophyll content (Castro and Sanchez‐Azofeifa 2008; Wang et al. 2018), moisture content (Koumbi‐Mounanga et al. 2015), and senescence (Castro and Sanchez‐Azofeifa 2008).

### 4.3 Leaf Spectra Show Plasticity in Response to Environmental Change

We found that population differences in cottonwood spectral phenotype are maximized in the wild, where both genetic and environmental differences can contribute, and attenuated but still distinct once environmental effects are removed by common growing conditions (Figure 3). This is consistent with our second hypothesis that environmental effects would modify leaf reflectance, and with previous research investigating environmental effects on genotype spectra between lab and field conditions (Li et al. 2023) and across island field sites (Seeley et al. 2023b). In some ways, this result is also similar to results from a previous study in oaks, where spectra could distinguish among populations growing in controlled watering conditions, as well as the environmental effect between two different watering treatments (Cavender‐Bares et al. 2016). However, that study did not find decreased accuracy when combining population replicates from multiple environments, which is in contrast with our results (Table 1: all gardens or at home). This difference may be because the environmental contrasts we investigated span a large gradient of 12°C in mean annual temperature, and the home sites vary in additional variables such as elevation, soils, interactions with insects, and access to groundwater (Bothwell et al. 2023). Thus, environmental variation among the three common gardens and among the home sites likely produced larger and more diverse effects on leaf reflectance than we would see from a watering treatment alone.

Across these disparate environments, genetically distinct populations adjusted their leaf spectra in diverse ways, producing gene x environmental interactions (Table 3), consistent with our second hypothesis. For example, the two coldest populations had similar reflectance at home and in the Cold common garden, although the similarity was highest in SWIR wavelengths for one population and NIR wavelengths for the other (Figure 4). Populations from slightly warmer locations such as BCEBUL and CLFLCR responded strongly to all common garden environments. Our most central population, CAFAUG, responded strongly to being transferred to the Hot common garden in the opposite direction than both colder and hotter source populations (Figure 4c). Using the spectral indices based on a few specific wavelengths, populations diverged in some garden environments while converging in others, but this pattern also varied across indices (Figure 5). For example, population divergence in greenness (NDVI) was highest in the Hot garden while divergence in water content (NDWI) was highest in the middle garden.

Importantly, despite all of these gene x environmental interactions we still achieved high accuracy (86%) for classifying populations when pooling trees from the three very different gardens (Table 1). We also found very high accuracy (100%) for distinguishing different common garden environments from each other (Table 1). This illustrates the potential power of hyperspectral data: multivariate analyses using the full spectra can correctly detect genetic and environmental influences despite numerous gene x environmental interactions affecting individual wavelengths or regions.

### 4.4 Using Leaf Reflectance to Interpret Plant Health Under Climate Change

One of the most pressing questions in global change biology is whether populations and genotypes will respond to their changing environments in ways that are beneficial or detrimental, decreasing or increasing their stress. For example, we have previously shown that phenological plasticity in Fremont cottonwood can allow populations to respond to increased growing temperatures by leafing out earlier and maximizing growth, but can also increase mortality from fall frost (Cooper et al. 2019). Thus, it is essential that we can detect whether trees in the wild are showing elevated stress when transplanted to new conditions or experiencing new climate extremes. One of the major benefits of hyperspectral data is that extensive work from agricultural and silvicultural applications has developed clear links between leaf reflectance data and indicators of abiotic (Sanaeifar et al. 2023) and biotic stress (Kuska et al. 2015; Haagsma et al. 2020). For example, hyperspectral data at leaf and/or canopy levels have been used to detect drought stress in maize (Weber et al. 2012; Cotrozzi et al. 2020), soybean (Guilherme Teixeira Crusiol et al. 2021), olive (Marino et al. 2014), and tomato (Alordzinu et al. 2021; Genangeli et al. 2023).

In *Populus* specifically, the Maximum Difference Water Index (MDWI) is a strong predictor of leaf water content among trees experiencing low versus high water stress (Eitel et al. 2006). Here, we found that MDWI decreased in the coldest populations moved to the Hot common gardens (Figure 5f). This could indicate that high elevation populations are less able to utilize the high amount of water needed for evaporative cooling at high temperatures (Moran et al. 2023; Posch et al. 2024) while still maintaining adequate water content. On the other hand, MDWI was one of the most variable indices across populations and genotypes (Figure 5f) and the weakest responder to temperature transfer distance from home conditions (Figure 6f). This suggests that more investigation of genetic variation in spectral responses to interactive effects of temperature and drought stress would be a valuable next step.

For spectral indicators of photosynthetic pigments and capacity, the environmental effects were much stronger and more consistent than those shown by MDWI. CI and sPRI indicated lower chlorophyll content and photosynthetic efficiency as populations were moved to the hot, southernmost common garden with a higher mean annual maximum temperature than their home sites (Figure 6, *R* ^2^ = 0.36–0.43). This is consistent with another recent study in *P. angustifolia*, where moving populations to cooler, higher latitude sites increased their chlorophyll content in apparent compensation for a reduced growing season (Kaluthota et al. 2024). These results are consistent with our third hypothesis, indicating negative plant health effects for genotypes transferred to environments with much hotter temperatures than those to which they are adapted (Grady et al. 2015; Cooper et al. 2019). Importantly, most previous studies on the effects of transfer distance have been limited to a few traits and plant performance outcomes such as growth or survival. Our results suggest hyperspectral data offer increased capacity to detect and anticipate the underlying phenotypic adjustments and stress responses that trees may be making as temperature and drought conditions increase across the Southwest. For example, failure to maintain hydraulic function necessary for leaf cooling at high temperatures has been connected to stand‐level mortality at the hot edge of the Fremont cottonwood species distribution (Moran et al. 2023). By monitoring spectral indices associated with temperature stress, such mortality events could be anticipated.

### 4.5 Using Leaf Reflectance Spectra for Conservation and Restoration Under Climate Change

Understanding differences in spectral phenotype between populations in changing environments is necessary to track tree acclimation and evolution in response to global change and make effective restoration and conservation decisions. For example, detecting genotypes and populations across a background of environmental variation allows us to use hyperspectral data to identify wild trees on the landscape (Robb et al. 2022). Using spectra, we can track range shifts of genetically distinct populations over time, assessing the effects of climate change and landscape connectivity on gene flow. This monitoring capacity is vital for regions like the American Southwest, where an ongoing megadrought since 2000 is considered the worst in 1200 years (Williams, Cook, and Smerdon 2022), and coincides with stand‐level diebacks in this and other tree species, resulting in changes in forest genetic structure (Sthultz, Gehring, and Whitham 2009). The distributions of both Fremont and narrowleaf cottonwood (*P. angustifolia*) are predicted to shift this century, with 50%–88% habitat loss depending on ecotype (Ikeda et al. 2017; Bothwell et al. 2021, 2023). Monitoring the fate of hot‐adapted versus cold‐adapted populations on the landscape can help us to develop better predictive models for the future distributions of these foundation species, whose loss would be devastating for diverse communities, sensitive and listed species, and riparian habitat (Durben et al. 2021).

In addition, the ability to infer both tree traits and tree performance from hyperspectral data suggests that we can use reflectance from wild trees of known genetic identity to assess both phenotypic plasticity and tree stress across populations. This could allow us to select and prioritize trees that maintain photosynthetic function across a wide range of conditions while continuing to effectively defend themselves from pathogens (Haagsma et al. 2020). Conversely, monitoring tree stress across the landscape may help to identify marginal habitats where restoration plantings are unlikely to succeed without substantial hydrological intervention, preventing wasted efforts in restoring marginal habitat that would be lost this century to climate change (Bothwell et al. 2021). Such large‐scale efforts may be further facilitated by translating leaf‐level results like those reported here into canopy‐level patterns that can be identified using hyperspectral spectrometers mounted on drones, aircraft, or satellites.

## 5 Conclusion

Landscape level mortality of forests worldwide is increasingly tied to drought and heat exposure (Allen et al. 2010). Regional drought led to increased mortality and decreased growth across a million hectares of *Populus tremuloides* in Canada (Hogg, Brandt, and Michaelian 2008) and forests in western North America, parts of the Amazon, and at the dry edges of species ranges may be consistently at elevated risk (Anderegg et al. 2019, 2022). Such challenges are also reflected by estimates that 30% of world's tree species are threatened with extinction (BGCI 2021). Our findings suggest that hyperspectral imaging can facilitate landscape level assessments of important tree traits that affect their mortality and survival. Using leaf‐level hyperspectral data from the same genetic identities across multiple common gardens and home sites, we show that leaf reflectance across the climate range of Fremont cottonwood is shaped by complex interactions between growing environment and genetic differences. Using spectra to interpret tree genetic identity or assess tree health in the wild requires careful attention to the possible roles of genetic and environmental influences. However, our findings suggest both of these objectives are possible due to the highly multivariate nature of the data and the growing wealth of research linking the interpretation of specific wavelengths to specific traits or indicators of plant health. With additional research validating those links between spectral phenotype and functional traits in this system, hyperspectral approaches should vastly improve our ability to monitor responses and design interventions associated with the rapid climate change already affecting forested landscapes across the Southwest.

## Conflicts of Interest

The authors declare no conflicts of interest.

## Supporting information

Supporting information.

## Data Availability

The data that support the findings of this study are available from EcoSIS at https://doi.org/10.21232/9bbY8fVJ.
