# Supplementary material for: Hyperspectral Leaf Reflectance Detects Interactive Genetic and Environmental Effects on Tree Phenotypes, Enabling Large‐Scale Monitoring and Restoration Planning Under Climate Change
Source: Plant Cell Environ. 2024 Nov 4;48(3):1842–57. doi: 10.1111/pce.15263 (PMC11788971; doi:10.1111/pce.15263)
Supplement: Supplementary file 1 — Supporting information. [file PCE-48-1842-s001.docx]

**Supplementary Information**

**Table S1**. Information for each home site and common garden, including location, elevation in meters above sea level and mean annual maximum temperature in Celsius (MAMT C°).

| **Name** | **Code** | **Lat** | **Long** | **Elevation (m)** | **MAMT C°** |
| --- | --- | --- | --- | --- | --- |
| Cold Common Garden |  | 38.09 | -109.59 | 1581 | 19.2 |
| Mid Common Garden |  | 34.26 | -112.07 | 982 | 25.2 |
| Hot Common Garden |  | 32.85 | -114.49 | 49 | 31.3 |
| Keams Canyon | KKHOPI | 35.81 | -110.17 | 1920 | 18.5 |
| Rattlesnake Canyon | MRNRAT | 34.78 | -111.61 | 1774 | 19.8 |
| Jack Rabbit, Little CO | JLAJAK | 34.96 | -110.44 | 1507 | 22.0 |
| Citadel Wash, Little CO | CLFLCR | 35.61 | -111.32 | 1299 | 23.2 |
| Sonoita Creek, Patagonia | PSASON | 31.54 | -110.76 | 1234 | 24.0 |
| San Pedro, Charleston | TSZSAN | 31.61 | -110.17 | 1219 | 24.1 |
| Willow Creek, Kingman | KWFWIL | 35.14 | -113.54 | 1126 | 25.2 |
| Clear Creek, Bullpen | BCEBUL | 34.54 | -111.70 | 1109 | 25.5 |
| Agua Fria, Horseshoe | CAFAUG | 34.26 | -112.07 | 988 | 26.2 |
| Santa Cruz, Tumacacori | TSETUM | 31.56 | -111.04 | 986 | 27.3 |
| Bill Williams, Colorado | LBWBIL | 34.28 | -114.06 | 143 | 30.5 |
| San Luis, Colorado | SCTMEX | 32.53 | -114.80 | 26 | 31.1 |

**Table S2.** The spectral indices used in this study, their common abbreviation, wavelength formula in nm, general application and references for each metric.

| Metric | Abbreviation | Formula | Use | Reference |
| --- | --- | --- | --- | --- |
| Chlorophyll Index | CI | $\frac{R750-R705}{R750+R705}$ | Chlorophyll content | Gitelson and Merzlyak 1994 |
| Carotenoid Reflectance Index | CRI | $\left( \frac{1}{R515}-\frac{1}{R565} \right)* R770$ | Carotenoids | Gitelson *et al.* 2006 |
| Photochemical Reflectance Index | PRI | $\frac{R531-R570}{R531+R570}$ | Photosynthetic activity | Gamon *et al.* 1992,  Penuelas *et al.* 1995 |
| Scaled Photochemical Reflectance Index | sPRI | $\frac{PRI+1}{2}$ | PRI positive values | Letts *et al.* 2008 |
| Normalized Difference Vegetation index | NDVI | $\frac{R800-R680}{R800+R680}$ | Greenness | Tucker 1979 |
| Normalized Difference Water Index | NDWI | $\frac{R860-R1240}{R860+R1240}$ | Canopy water | Gao 1996 |
| Maximum Difference Water Index | MDWI | $\frac{{(R}_{max}1500:1750)-{(R}_{min}1500:1750)}{{(R}_{max}1500:1750)+{(R}_{min}1500:1750)}$ | Water status, Water stress | Eitel *et al.* 2006 |

**Table S3.** Genetic x environmental interaction statistics for all spectral indices. Pop = population, PopGeno = genotype within population. Significant effects at p < 0.05 are shown in bold font.

| **Index** | **Effect** | **F** | **Df** | **Pr(>F)** | **R^2^** |
| --- | --- | --- | --- | --- | --- |
| CI | **Garden** | 54.6 | 2,17.75 | **<0.001** | 0.34 |
| CI | **Pop** |  | 1 | 0.492 |  |
| CI | **PopGeno** |  | 1 | **<0.001** |  |
| CI | **Pop x Garden** |  | 1 | **<0.001** |  |
| CI | PopGeno x Garden |  | 1 | 1.000 |  |
| CRI | **Garden** | 10.9 | 2,17.87 | **0.001** | 0.16 |
| CRI | Pop |  | 1 | 0.206 |  |
| CRI | **PopGeno** |  | 1 | **0.013** |  |
| CRI | **Pop x Garden** |  | 1 | **<0.001** |  |
| CRI | PopGeno x Garden |  | 1 | 1.000 |  |
| sPRI | **Garden** | 59.1 | 2,19.01 | **<0.001** | 0.51 |
| sPRI | Pop |  | 1 | 1.000 |  |
| sPRI | PopGeno |  | 1 | 0.498 |  |
| sPRI | **Pop x Garden** |  | 1 | **<0.001** |  |
| sPRI | PopGeno x Garden |  | 1 | 1.000 |  |
| NDVI | **Garden** | 12.6 | 2,18.03 | **<0.001** | 0.19 |
| NDVI | Pop |  | 1 | 0.234 |  |
| NDVI | PopGeno |  | 1 | 0.313 |  |
| NDVI | **Pop x Garden** |  | 1 | **<0.001** |  |
| NDVI | PopGeno x Garden |  | 1 | 1.000 |  |
| NDWI | **Garden** | 16.0 | 2,17.72 | **<0.001** | 0.17 |
| NDWI | Pop |  | 1 | 0.253 |  |
| NDWI | PopGeno |  | 1 | 0.070 |  |
| NDWI | **Pop x Garden** |  | 1 | **<0.001** |  |
| NDWI | PopGeno x Garden |  | 1 | 1.000 |  |
| MDWI | Garden | 1.6 | 2,18.80 | 0.269 | 0.03 |
| MDWI | Pop |  | 1 | 0.993 |  |
| MDWI | **PopGeno** |  | 1 | **0.021** |  |
| MDWI | **Pop x Garden** |  | 1 | **<0.001** |  |
| MDWI | PopGeno x Garden |  | 1 | 1.000 |  |

**Figure S1. Mean reflectance amplitudes for each population differ across environments.** Panels are arranged from coldest to hottest Mean Annual Maximum Temperature (MAMT) of the population’s home site. For each population, the colored lines represent the average reflectance of leaves surveyed in the home site and in each common garden. The three hottest populations do not have data for the cold common garden due to mortality. We were unable to access the coldest (KKHOPI) and hottest (SCTMEX) home sites due to travel restrictions.

**
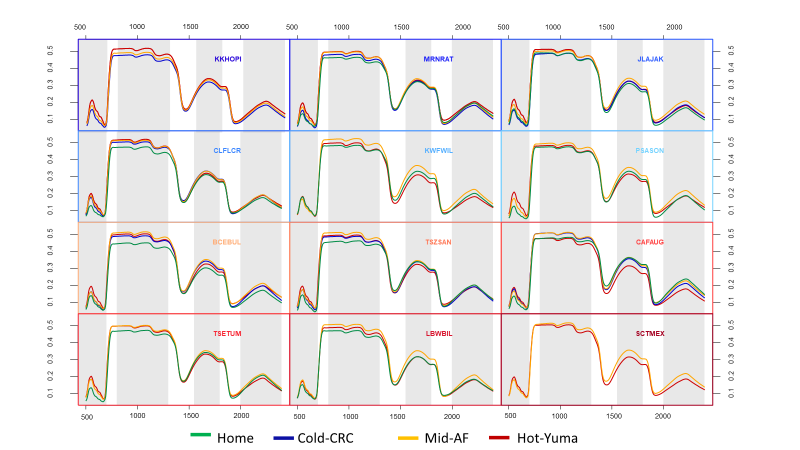
**
